# Successful Use of the Er,Cr:YSGG Laser for Gingival Depigmentation

**DOI:** 10.7759/cureus.72867

**Published:** 2024-11-02

**Authors:** Rana Almusallam, Maha Ali Al-Mohaya

**Affiliations:** 1 Oral Medicine, Prince Sultan Military Medical City, Riyadh, SAU; 2 Oral Medicine and Special Care Dentistry, Prince Sultan Military Medical City, Riyadh, SAU

**Keywords:** ablation, cr:ysgg, depigmentation, er, gingival, hyperpigmentation

## Abstract

The color of the gingiva is determined by the degree of vascularization, epithelial thickness, and the number of melanin pigments within the epithelium. Melanin pigmentation is caused by abnormal or increased deposition of melanin by active melanocytes located mainly in the basal and supra-basal cell layers of the oral epithelium. Oral melanin pigmentation occurs most frequently in the gingiva, known as gingival hyperpigmentation (GHP), which is a common esthetical concern with variable etiologic factors. Clinically, GHP reveals asymptomatic, well-defined, generalized or localized, light-to-dark brown macules of variable sizes. Gingival depigmentation (GD) is a procedure used to remove or reduce excessive pigmentation using several therapeutic approaches, which include surgical, chemical, electrosurgical, and cryosurgical procedures. Many clinicians prefer to use laser ablations such as CO_2_, diode, Er:YAG, and Er,Cr:YSGG, which can be utilized safely and effectively without postoperative discomfort. Er,Cr:YSGG with a 2780 nm wavelength has an ablative mechanism of action with high water absorption. Herein, we present a case of a 16-year-old girl with a darkly pigmented gingiva. Clinical examination revealed generalized physiologic pigmentation in the maxillary and mandibular anterior gingiva. Treatment was carried out by using an Er,Cr:YSGG laser including a wavelength of 2780 nm, a frequency of 50 Hz, 2.5 W, 10-20% water, and 20-40% air, employing the MZ8 tip in contact mode localized only on pigmentated areas. The ablated regions healed almost completely in two weeks without pain or discomfort, and the color was comparable to the normal gingiva. In the six-month follow-up, the patient was seen with full satisfaction. GD by the Er,Cr:YSGG laser was found to be a safe and efficient approach.

## Introduction

The normal physiologic color of the gingiva is coral or salmon pink; however, it may range from pale pink to dark bluish-purple. This physiological variation is determined by multiple factors involving the degree of vascularization, epithelial thickness, quantity of keratinization, and the amount of melanin pigments within the epithelium [1]. Melanin, carotene, oxyhemoglobin, and reduced hemoglobin are the primary pigments contributing to the normal color of the oral mucosa [2,3]. Melanin pigmentation is produced through increased deposition of melanin and by active melanocytes located in the basal and suprabasal cell layers of the oral epithelium [2].

The color of the oral melanin pigmentation may differ from light-to-dark brown or black, depending on the aggregation of melanin in the tissue that occurs most frequently in the gingiva, known as gingival hyperpigmentation (GHP) or "black gums." GHP is a common esthetical concern with variable etiologic factors such as physiologic pigmentation, smoking, and medications such as tetracyclines and antimalarial drugs. Furthermore, there are manifestations of systemic diseases such as Addison's disease and Peutz-Jeghers syndrome. Clinically, GHP reveals asymptomatic, well-defined, generalized or localized, light-to-dark brown macules of variable sizes [1,2].

This problem worsens in patients with a "gummy smile" or excessive gingival display while smiling or talking, mainly by fair-skinned people who frequently request cosmetic therapy [4]. Gingival depigmentation (GD) is a procedure used to remove or reduce excessive pigmentation using several therapeutic approaches, which include surgical, chemical, electrosurgical, and cryosurgical procedures [1,2,4].

Although the selection of a technique is mainly based on clinical practice and individual preferences [1], many clinicians prefer to use laser ablation such as CO_2_, diode, Er:YAG, and Er,Cr:YSGG [1]. This article illustrates that the Er,Cr:YSGG laser can be utilized safely and effectively, resulting in pleasant, significant improvement of gingival discoloration and rapid wound healing without postoperative discomfort [5].

## Case presentation

**Figure 1:**
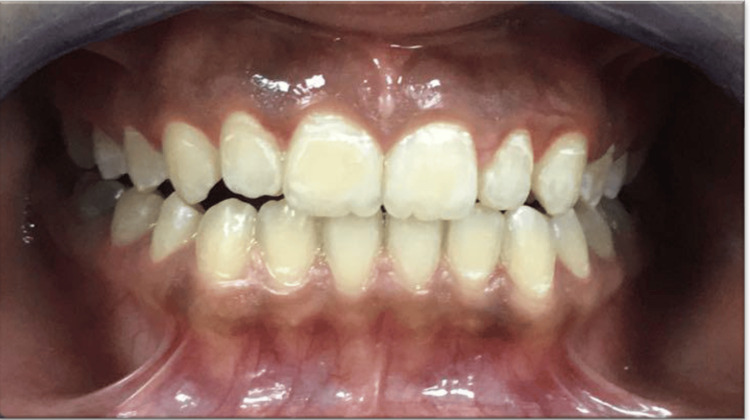
The preoperative image revealed generalized physiologic pigmentation in the maxillary and mandibular anterior gingiva.

A 16-year-old medically fit girl presented to the oral medicine clinic complaining of a darkly pigmented gingiva. Based on a detailed review of the patient's medical and family history, no systemic diseases were reported, and the extraoral examination was non-contributory. The intraoral clinical examination revealed generalized physiologic pigmentation in the maxillary and mandibular anterior gingiva (Figure 1).

Informed consent was obtained for the procedure and photograph. The pigmented areas were divided into four regions, and depigmentation was performed in two regions in each visit, commencing with the anterior region.

**Figure 2:**
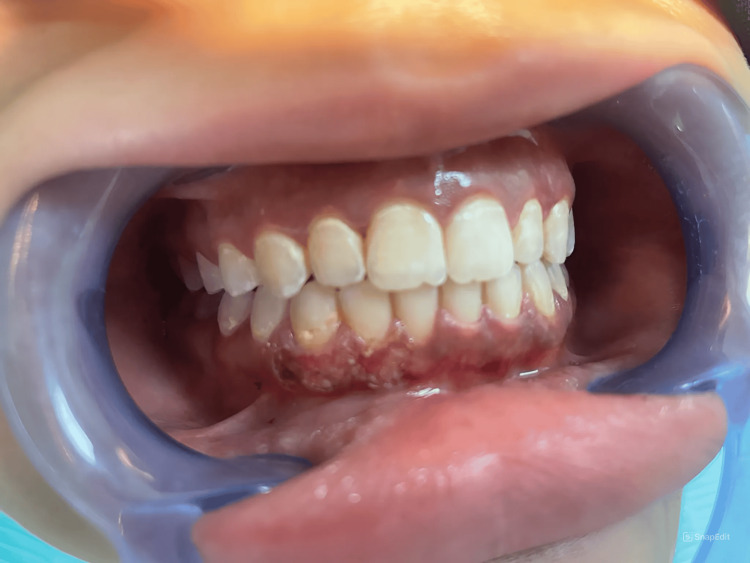
Intraoperative laser ablation involving the interdental papilla was established from the mucogingival junction toward the free gingival margin.

Treatment was carried out after infiltration of lidocaine 2% with 1:80,000 epinephrine for local anesthesia, using an Er,Cr:YSGG laser with a wavelength of 2780 nm, a frequency of 50 Hz, 2.5 W, 10-20% water, and 20-40% air, employing the MZ8 tip in contact mode localized only on pigmentated areas. Laser ablation involving the interdental papilla was established from the mucogingival junction toward the free gingival margin. The laser beam was utilized using the "brush technique," and the tip remained in motion (Figure 2).

The treatment was finished in approximately 15 minutes for every session. After completion of each procedure, remnants of the ablated tissue were removed using a sterile gauze dampened with saline solution. All laser safety measures were implemented throughout the procedure. No periodontal pack or additional material was applied to support the healing process. Postoperative instructions were given to the patient to avoid vigorous rinsing and spicy hard or hot foods for the first couple of days and rinse with warm saline after surgery. Analgesics were prescribed as needed.

**Figure 3:**
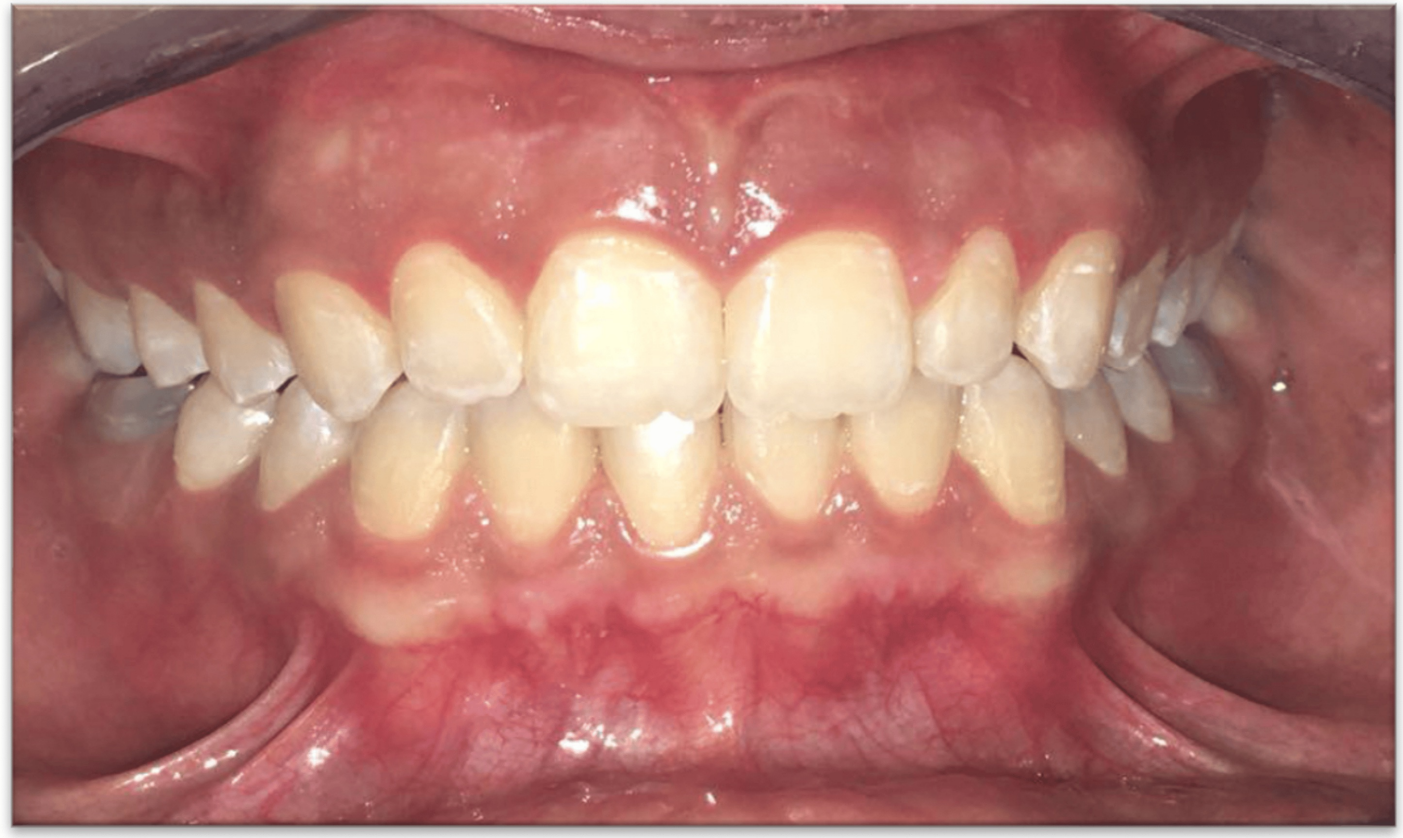
Normal gingival coloration two months postoperatively.

After 24 hours, the gingiva was coated with a layer of fibrin. The ablated area healed in approximately two weeks without pain or discomfort, and its color was comparable to that of normal gingiva. The patient was evaluated after two months (Figure 3) with complete satisfaction. Furthermore, during the six-month follow-up, the patient was seen with complete satisfaction. She is scheduled for a one-year follow-up for re-evaluation and assessment of re-pigmentation.

## Discussion

Physiologic pigmentation of the gingiva is clinically demonstrated as multiple or single melanin pigmentation with varying quantities in divergent ethnic groups and races. It is a common esthetic concern that might have a significant psychological impact on individuals [2,5].

Intraorally, the gingivae are the most common area for physiological melanin pigmentation; this is associated with the high number of melanocytes existing in the gingiva [3,5]. GD is a therapeutic approach used to remove the melanin hyperpigmentation. Different techniques have been utilized for this purpose, including mechanical, surgical, electrosurgical, cryosurgical, free gingival grafts, and lasers [2,6].

Surgical depigmentation involves eliminating the gingival epithelium and a layer of connective tissue. It may cause alveolar bone loss, prolonged healing, pain, excessive bleeding, tissue destruction, and swelling. It also requires high technical skills and special instruments [1,7]. The scalpel surgical procedure was one of the earliest methods for GD and is a gold-standard treatment modality [8].

Laser therapy is another effective treatment option for hyperpigmented epithelium. Various dental lasers have been suggested for the successful treatment of GHP. The results of laser therapy depend on the type of laser and the type of tissue. The wavelength and tissue type determine the laser-tissue interaction, as the tissue can transmit, scatter, reflect, or absorb laser light [9].

The depth of penetration of a laser in biological tissue is dependent on its wavelength. Thus, lasers are classified into two types depending on their wavelength. These lasers include heat-producing lasers of an intensely penetrating type, where the laser light penetrates and scatters into the tissue more deeply, such as carbon dioxide (CO_2_) and Nd:YAG lasers, semiconductor diode lasers, and argon lasers. The second type is non-heat-producing lasers, where the laser light is absorbed in the superficial layer and does not penetrate deeply, e.g., Er:YAG and Er,Cr:YSGG lasers [5,9,10].

Chromophores, which are composed of melanin, pigmented proteins, hemoglobin, hydroxyapatite, and water, absorb the energy of laser photons in the oral cavity.

When the laser photon energy is absorbed through the water in the cells, the temperature increases, generating micro-explosions in a process termed water-induced ablation, which influences the gingival epithelium. When the laser photon energy is absorbed by the melanin or hemoglobin chromophores, the laser generates a thermal reaction involving heat accumulation, which is subsequently detectable as coagulation, denaturation, vaporization, necrosis, and carbonization [10].

Er,Cr:YSGG with a 2780 nm wavelength has an ablative mechanism of action with high water absorption, followed by vaporization and thermomechanical micro-explosion in the cells [3,11]. Compared to diode lasers, Erbium lasers induce less thermal damage, resulting in ablation without carbonization and minimal heating. This leads to minimal thermal damage to the underlying tissues and promotes expedited wound healing after irradiation [2,6,11]. The laser ablates the target tissue elements while providing a dry field of surgery by sealing small blood and lymphatic vessels, resulting in reduced postoperative edema and pain reduction by sealing the end of sensory nerves compared to conventional surgical techniques [12].

However, the laser approach demonstrates superior results in terms of reduced postoperative pain [8]. Laser application is preferable for quick healing, less scar tissue formation, and bactericidal effects [1,4,5]. Nevertheless, inappropriate application and settings may cause gingival recession and damage to the periosteum and bone, causing pain and delayed healing [7].

All wavelengths can effectively and efficiently eliminate gingival melanin pigmentation. Furthermore, their mode of action and the effect of surrounding structures were variable. Considering the depigmentation outcome, cell regeneration, postoperative comfort, and re-pigmentation tendency, it is essential to recognize a balance between the parameters of the treated tissue and the processes involved in laser-tissue effects [10].

A review article by Bakhshi et al. has reported a rapid healing effect for the erbium laser family, particularly Er,Cr:YSGG [6]. Re-pigmentation after GD is an important point of which clinicians should be aware. Re-pigmentation has been reported following the use of different techniques [2,11].

## Conclusions

Er,Cr:YSGG laser radiation has been identified to be a safe and efficient therapeutic approach for GHP. Postoperative patient satisfaction was optimum. The gingiva healed and regenerated without complications, such as infection, pain, swelling, or scarring. No re-pigmentation occurred during the short follow-up period; however, a long follow-up is highly recommended.
